# Conditional cash transfers to improve use of health facilities by mothers and newborns in conflict affected countries, a prospective population based intervention study from Afghanistan

**DOI:** 10.1186/s12884-019-2327-2

**Published:** 2019-06-03

**Authors:** Karen M. Edmond, Abo Ishmael Foshanji, Malalai Naziri, Ariel Higgins-Steele, Jane Machlin Burke, Natalie Strobel, Farhad Farewar

**Affiliations:** 1 UNICEF Afghanistan, UNOCA, Jalalabad Road, Kabul, Afghanistan; 2 Ministry of Public Health, Health Economics and Financing Directorate, Kabul, Afghanistan; 3 Maternal and Child Health Consultant, Beirut, Lebanon; 4 School of Paediatrics and Child Health, University of Western Australia, Perth, Australia

**Keywords:** Maternal and newborn health, Inequalities, Cash transfer

## Abstract

**Background:**

The effects of conditional cash transfer (CCT) programs on maternal and child health (MCH) service use in conflicted affected countries such as Afghanistan are not known.

**Methods:**

We conducted a non-randomised population based intervention study in six Afghanistan districts from December 2016 to December 2017. Six control districts were purposively matched. Women were eligible to be included in the baseline and endline evaluation surveys if they had given birth to one or more children in the last 12 months.

The intervention was a CCT program including information, education, communication (IEC) program about CCT to community members and financial incentives to community health workers (CHWs) and families if mothers delivered their child at a health facility. Control districts received standard care.

The primary objective was to assess the effect of CCT on use of health facilities for delivery. Secondary objectives were to assess the effect of CCT on antenatal care (ANC), postnatal care (PNC) and CHW motivation to perform home visits.

Outcomes were analysed at 12 months using multivariable difference-in-difference linear regression models adjusted for clustering and socio demographic variables.

**Results:**

Overall, facility delivery increased in intervention villages by 14.3% and control villages by 8.4% (adjusted mean difference [AMD] 3.3%; 95% confidence interval [− 0.14 to 0.21], *p* value 0.685). There was no effect in the poorest quintile (AMD 0.8% [− 0.30 to 0.32], p value 0.953). ANC (AMD 45.0% [0.18 to 0.72] p value 0.004) and PNC (AMD 31.8% [− 0.05 to 0.68] p value 0.080) increased in the intervention compared to the control group. CHW home visiting changed little in intervention villages (− 3.0%) but decreased by − 23.9% in control villages (AMD 12.2% [− 0.27 to 0.51], p value 0.508). CCT exposure was 27.3% (342/1254) overall and 10.2% (17/166) in the poorest quintile.

**Conclusions:**

Our study demonstrated that a CCT program provided to women aged 16–49 years can be implemented in a highly conservative conflict affected population. CCT should be scaled up for the poorest women in Afghanistan.

## Background

There have been considerable improvements in maternal and newborn health across Asia [1, 2]. However, inequities in access and use of maternal and newborn health services remain widespread. Barriers include: cost, distance, perceived quality of care and poor awareness of healthcare options [1, 2]. ‘Demand side financing’ has been used for many years to incentivise families to use primary care services [3]. The type of scheme varies from unconditional transfers (e.g. money provided following the birth of a child), vouchers (reimbursable for a range of services) and conditional cash transfer (CCT) schemes (payment for specific services) [4].

Many low and middle income countries have implemented CCT schemes. Two systematic reviews have examined impact on use of maternal and child health services in the last 10 years including studies from 17 countries [5, 6]. The authors of the systematic reviews concluded that CCT can have important short term impacts on antenatal care and facility delivery but that effects are dependent on family ‘exposure’ and awareness of the scheme, access to transport, efficiency of payments and staffing in health facilities [5, 6]. Monitoring of non-incentivised services is also important as coverage of non incentivised services may decrease as more attention is provided to incentivised services. However, to date there appears to be only one small study from India which specifically assessed the effects of CCT on non incentivised maternity services (antenatal care [ANC] and postnatal care [PNC]) [7].

No CCT studies also appear to have been conducted in conflict affected areas, though one study in Afghanistan has assessed feasibility [8]. We recently reported that barriers to MCH use in Afghanistan are similar to other Asian countries but lack of decision making ‘power’ of women in the family and the presence of anti-government elements in small villages are also important determinants [9, 10]. Families in conflict affected countries such as Afghanistan are likely to require additional assistance to enable them to use financial incentives. This includes community level support [11].

Thus, in 2015, the Ministry of Public Health (MoPH) in Afghanistan in partnership with Unicef, developed a CCT intervention to assist rural Afghanistan women to access health facilities for maternal and newborn health services in rural areas.

The primary objective of this study was to assess the effectiveness of the MoPH CCT intervention on use of health facilities for delivery. Secondary objectives were to assess effects on (i) maternal care seeking for non-incentivised services such as ANC and PNC and (ii) community health worker (CHW) motivation to perform home visits. In addition we assessed effects in the poorest and least poor quintiles.

## Methods

### Design

This was a non-randomised, population based intervention study conducted over a 12 month period from November 2016 to December 2017. The intervention was implemented from December 2016 to December 2017. For the evaluation the baseline survey was conducted over a 1 month period in November 2016 and the endline survey was conducted in December 2017. All pregnant women aged 16 years and above who were resident in the study districts were eligible to receive the intervention. Women were only eligible to be included in the baseline and endline surveys if they were postpartum and had given birth to one or more children in the last 12 months.

### Setting

Afghanistan has a population of 32 million people and is mountainous and landlocked. It is prone to natural disasters including avalanches, earthquakes and flooding [12, 13]. Many roads become in accessible in winter seasons. Conflict and violence has increased over the last two years [14]. Although access to health facilities has improved over the last fifteen years, in 2016 over 50% of families had to travel two or more hours to reach a primary health care centre [15].

In Afghanistan, the basic health system includes district hospitals, health centres and outreach services including vaccination teams. There are also volunteer CHWs based at ‘health posts’ (usually the CHW home) in each village. The CHWs are trained to provide basic health care such as health promotion advice, referral of sick women and children and information, education, communication (IEC) for preventive services such as immunisation, ANC, PNC and facility delivery. They also provide basic medicines such as iron and folic acid, antibiotics for pneumonia and oral rehydration therapy (ORS) for diarrhoea. Their basic training includes intensive preservice training over a three month period and yearly one week refresher training. Each CHW is supervised by a paid Ministry community health supervisor (CHS). The CHS provides supportive supervision, monitoring and reporting functions [16, 17]. 5% of health facilities provide private fee paying services. The remaining 95% are public facilities providing free services including MCH [16]. More details are presented in Appendices 1 and 2.

**Fig. 1:**
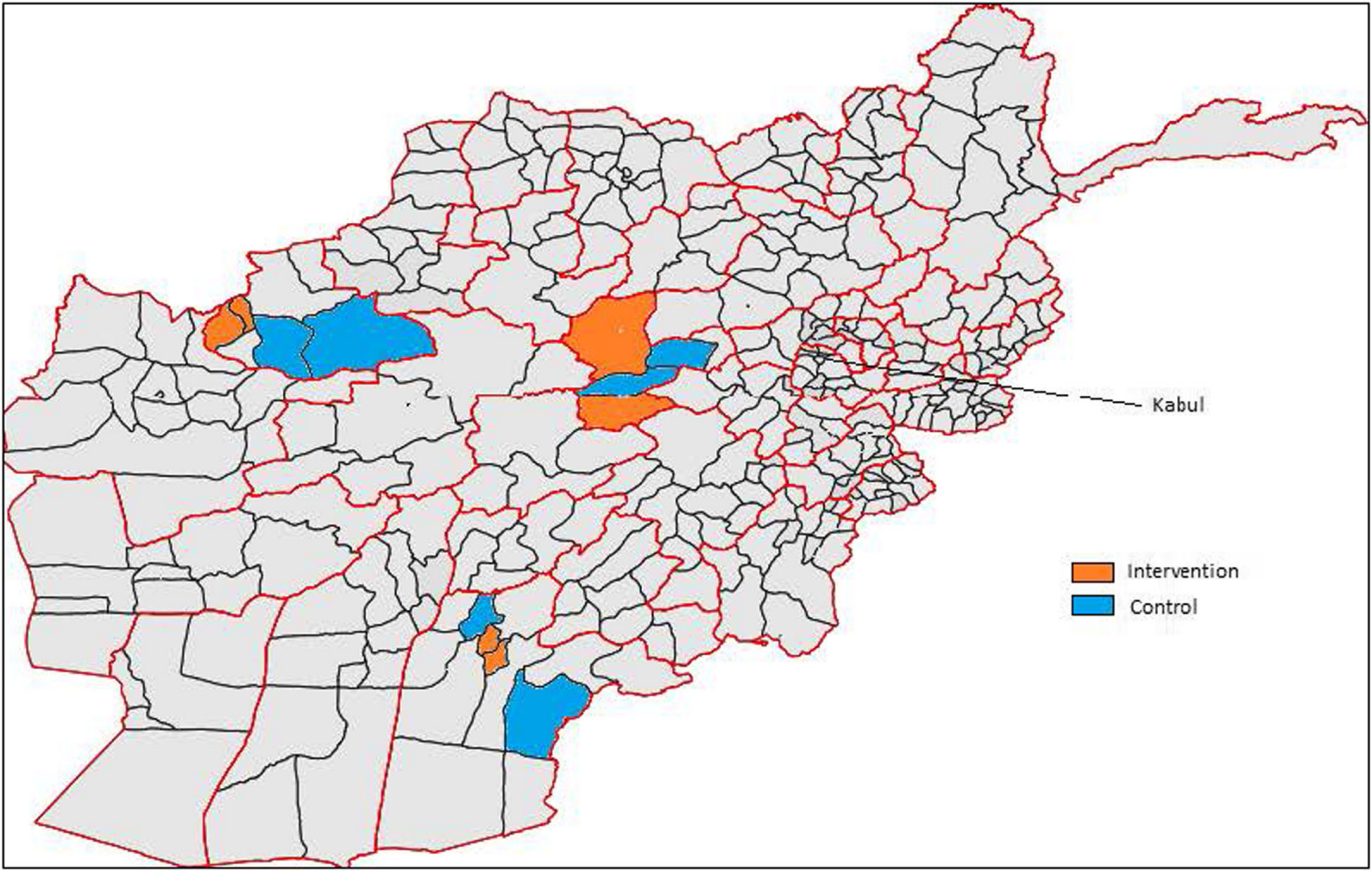
Map of Afghanistan. (https://commons.wikimedia.org/wiki/File:Blank_map_of_Afghanistan_districts.svg) [18] with amendments to show geographic distribution of the intervention and control districts

This project was based in three provinces of Afghanistan (Badghis, Bamyan and Kandahar). The project team purposively selected two intervention districts per province to ensure conflict affected areas and geographically hard to reach areas were represented (Badghis [Abkamary, Moqur], Bamyan [Waras, Kahmard], and Kandahar [Dand, Daman]). Two control districts per province were then chosen with similar socio demographics, access, clinic density and security (Badghis [Qades, Jawand], Bamyan [Punjab, Saighan], Kandahar [Arghandaab, Spin Boldak]). Figure 1 displays the map of the study area. Table 1 and Appendix 1 display profile data for the study area.

**Table 1:** Sociodemographic characteristics of women included in the study compared between intervention and control villages

	Number (%) of mothers in intervention villages		Number (%) of mothers in control villages	
	Baseline	Endline	Baseline	Endline
	(*n* = 1199)	(*n* = 1254)	(*n* = 1242)	(*n* = 1237)
Province				
Bamyan	421 (35.1%)	403 (32.1%)	413 (33.3%)	403 (32.6%)
Badghis	387 (32.3%)	400 (31.9%)	412 (33.2%)	404 (32.7%)
Kandahar	391 (32.6%)	451 (36.0%)	417 (33.6%)	430 (34.8%)
Maternal education				
Primary school (year 1–6)	38 (3.2%)	46 (3.7%)	74 (6.0%)	86 (7.0%)
Secondary school (year 7–9)	33 (2.8%)	34 (2.7%)	40 (3.2%)	38 (3.1%)
High school (year 10–12)	32 (2.7%)	72 (5.7%)	57 (4.6%)	73 (5.9%)
Advanced education (year 12+)	12 (1.2%)	20 (1.6%)	22 (1.8%)	49 (4.0%)
No education	1082 (90.2%)	1057 (84.3%)	1038 (83.6%)	983 (79.5%)
Not known	0 (0.0%)	25 (2.0%)	11 (0.9%)	8 (0.7%)
Maternal literacy				
Literate	223 (18.6%)	412 (32.8%)	279 (22.5%)	382 (30.9%)
Not literate	976 (81.4%)	842 (67.2%)	963 (77.5%)	855 (69.1%)
Mother’s age				
16–24 years	469 (39.1%)	496 (39.6%)	455 (36.6%)	391 (31.6%)
25–29 years	407 (33.9%)	307 (24.5%)	346 (27.9%)	386 (31.2%)
30–39 years	213 (17.8%)	364 (29.0%)	145 (11.7%)	282 (22.8%)
40 years or older	108 (9.0%)	86 (6.9%)	70 (5.6%)	47 (3.8%)
Not known	2 (0.2%)	1 (0.1%)	226 (18.2%)	131 (10.6%)
Mother’s parity				
1	112 (9.3%)	138 (11.0%)	96 (7.7%)	188 (15.2%)
2	161 (13.4%)	165 (13.2%)	183 (14.7%)	238 (19.2%)
3	209 (17.4%)	160 (12.8%)	242 (19.5%)	208 (16.8%)
4	174 (14.5%)	203 (16.2%)	209 (16.8%)	150 (12.1%)
5 or more	543 (45.3%)	588 (46.9%)	512 (41.2%)	453 (36.6%)
Wealth quintile				
Quintile 1 (poorest)	111 (9.3%)	166 (13.2%)	358 (28.8%)	258 (20.9%)
Quintile 2	223 (18.6%)	255 (20.3%)	228 (18.4%)	169 (13.7%)
Quintile 3	294 (24.5%)	201 (16.0%)	165 (13.3%)	223 (18.0%)
Quintile 4	241 (20.1%)	200 (16.0%)	220 (17.7%)	222 (18.0%)
Quintile 5 (least poor)	239 (19.9%)	270 (21.5%)	212 (17.1%)	152 (12.3%)
Missing	91 (7.6%)	162 (12.9%)	59 (4.8%)	213 (17.2%)
Access to any health facility				
Less than 30 mins	422 (35.2%)	419 (33.4%)	299 (24.1%)	376 (30.4%)
30 mins to 1 h	405 (33.8%)	509 (40.6%)	595 (47.9%)	531 (42.9%)
1–2 h	172 (14.4%)	236 (18.8%)	183 (14.7%)	211 (17.1%)
2 h to half a day	124 (10.3%)	65 (5.2%)	25 (2.0%)	35 (2.8%)
More than half a day	29 (2.4%)	1 (0.1%)	10 (0.8%)	54 (4.4%)
Not known	47 (3.9%)	24 (1.9%)	130 (10.5%)	30 (2.4%)

### CCT intervention

The intervention was a CCT program with three key elements: (i) information, education, communication (IEC) program about CCT to community members; (ii) financial incentives to CHWs if they visited pregnant women at home and assisted them to travel to health facilities for delivery; (iii) financial incentives to mothers if they delivered their child at a health facility. The CCT program was based on prior intensive formative research [9]. There were no private birthing facilities in the study area thus the intervention only involved public facilities.

The IEC program focused on four key issues: the importance of health facility delivery, the resources (including CHWs) available to assist with travel to health facilities for delivery, the mother’s cash incentive for health facility delivery and the CHW’s cash incentive if he or she facilitated referral. The IEC program used four delivery channels: billboards, brochures, CHWs and village leaders. A dedicated graphic designer developed and pretested key messages for billboards and brochures which were displayed in each village and each health facility. In November 2016 all CHWs from each village in the intervention districts and their supervisors (CHSs) received a one day training course in the CCT intervention, especially the use of the CCT IEC material and key issues (the importance of health facility delivery, the resources [including CHWs] available to assist with transfer to health facilities for delivery, the mother’s cash incentive if she delivered at health facility and the CHWs cash incentive if they facilitated referral). They were also trained in the importance of providing home visits and visiting disadvantaged hard to reach families, including fathers, male heads of households and women of reproductive age. They were also trained in CCT referral record keeping. Village leaders also received a similar one day training course in November 2016. After consultation with key MoPH stakeholders, the importance of ANC and PNC services was also included in all training programs and IEC messaging.

The mother received a cash transfer of 1000 Afghani (approximately USD $15) if she delivered at a health facility regardless of the transport mechanism she chose. CHWs received a cash transfer of 300 Afghani (approximately USD $5) if they accompanied the mother to the health facility or if the mother provided a CHW referral slip when she arrived for delivery. Funds were provided to both mother and CHW at the health facility before the mother was discharged home.

### Control areas

The control areas in Badghis [Qades Jawand], Bamyan [Punjab Saighan] and Kandahar [Arghandaab, Spin Boldak]) received standard care and did not receive our CCT intervention. Health facilities in control and intervention districts were provided with a health system strengthening (HSS) package over 24 months prior to the intervention delivery and throughout the intervention period. This included the UNICEF/WHO training package in essential newborn care (ENC) and Basic and Emergency Obstetric and Newborn Care (BEmONC). Women did not have to pay for delivery at any facilities in the control areas.

### Definitions

The primary outcome measure was the proportion of women who delivered in a health facility in the study area.

Secondary outcome measures were: the proportion of women who received at least one ANC visit, proportion of women who received at least one PNC visit and the proportion of women who received at least one CHW home visit.

A composite measure of ‘treatment’ (or ‘exposure’) (i.e. receipt of the intervention) was defined as a mother who reported that she knew (i) there was any program that would give her money if she delivered in a health facility or (ii) she would receive our CCT intervention if she delivered in a health facility. A composite measure of ‘non treatment’ was also defined as a mother who reported that she did not know (i) or (ii). We did this because there are many programs in Afghanistan which provide financial incentives for health care and we felt in our study area mothers are not likely to know which particular ‘organization’ is providing the funds. We wanted to be careful to capture all women who felt they would receive any funds, as we felt that was the most accurate representation of ‘exposure’. We considered that women who answered ‘yes’ to (ii) should also answer ‘yes’ to (i). However we felt that women might not understand question (i) so we thought it would be most conservative to include (ii) as well into the composite measure as a combined response. The questions referred to any births (previous or future). Other study definitions are presented in Appendix 2.

### Data collection

We randomly selected households to be visited for baseline and endline data collection in each district using a standard two stage sampling method with probability of selection proportional to size (i.e. random selection of villages followed by random selection of households) [19, 20]. Use of the same cooking hearth was used to define a household. Villages in Afghanistan do not use household listings, so we selected survey respondents using the ‘random walk’ method [21]. This method involved enumerators starting at a community landmark, spinning a bottle, walking in the direction the bottle points, and stopping at every third household to select a survey respondent.

Baseline and endline data were collected from women who were less than 12 months postpartum using a standardised structured survey questionnaire. Survey domains included self reported: education level, ownership of specific assets, sanitation facilities; service use for ANC, delivery, PNC; any home visits received from CHWs; and knowledge of the CCT intervention as defined above.

A separate independent team of female data collectors were trained over a five-day period and were supervised by two independent data supervisors. The data supervisors performed spot (unscheduled) checks of approximately 10% of the surveys together with a member of the study team. An additional 10% of surveys were selected for repeat survey. The data supervisors reviewed each survey form. Forms were not submitted for data entry until they were considered complete and without error. An additional random subsample of 10% of survey forms were also reviewed in the first week of data collection.

### Data analysis

For the outcome evaluation we assessed mean differences in key variables (delivery in a health facility), from baseline (data collection immediately prior to the intervention) to endline (data collection 12 months after the intervention commenced) and compared effects in the intervention areas compared to control areas.

We calculated that we required a sample size of 3395 women to provide 90% power at a 5% significance level for the primary outcome [22]. These calculations were based on recent data at the provincial level, which indicated that there would be a baseline level of facility delivery of 50%, and previous studies that indicated that there would be at least a 25% change in service use due to the intervention [5, 16]. We also assumed that there would be one eligible woman per household. We calculated that this sample size would provide adequate power for the secondary outcomes as well.

We used the multivariable “difference-in-differences” (DiD) approach to estimate the mean effect of the intervention on each outcome [23, 24]. The “difference-in-differences” approach is based on comparing mean differences in the intervention group (before and after the intervention) to mean differences in the control group (before and after the intervention) and assumes that trends in both groups are the same in the absence of the intervention. We also assessed two key DiD assumptions (i) parallel trends in the primary and secondary outcomes pre and post intervention (using additional data from the Health Management Information System (HMIS) from 2013 to 2014) and (ii) stable distribution of covariates between intervention and control groups from baseline to endline [23–25].

Principal components analysis was used to create the wealth quintiles using standard methods [26]. Multivariable linear regression models were constructed to adjust for clustering by district and potential confounders decided a priori (maternal age, parity, education, quintile, access to health facilities) and to calculate adjusted mean differences (AMD), 95% confidence intervals (95% CI) and corresponding *p* values.

In addition, we performed a ‘treatment on the treated’ analysis with a restricted sample [27]. In this analysis intervention women were restricted to only women who were “treated” (i.e. who received the intervention composite measure as defined above). Control women were restricted to only women who did not receive the intervention (i.e. who were “not treated”). The restrictions were applied to endline mothers only. The restrictions were not applied to baseline mothers because baseline mothers had not been exposed to the intervention. Stata version 14.2 was used for all analyses.

## Results

### General characteristics

Of 2540 women aged 16 years and above invited to participate in the baseline evaluation survey, 2441 agreed to participate (96.1%). Of 2505 women approached at endline 2491 agreed to participate (99.4%). A total of 4929 women were recruited, 2453 intervention (1199 baseline, 1254 endline) and 2476 control (1242 baseline 1234 endline) (Table 1). 33.1% (1632) were from Bamyan, 32.2% (1588) from Badghis, and 34.3% (1681) from Kandahar. 36.7% (1811) women were aged 16–24 years, 10.8% (534) had only one child, 84.4% (4160) had no education, 534 (10.8%) had only one child, and 893 (18.1%) were in the poorest quintile (Table 1), Overall, 10.9% (525/4929) of mothers had missing data for quintile and 360/4929 (7.1%) had missing data for maternal age.

### Exposure

The composite measure of CCT program exposure (mother reported that she knew (i) there were any programs that would give her money if she delivered in a health facility or (ii) she would receive our CCT incentive if she delivered in a health facility) was low overall 342/1254 (27.3%) (Table 2). (17.9% (224) knew only (i) (that there were any programs that would give a mother money) plus 4.5% (57) knew only (ii) (about our CCT program) plus 4.9% (61) knew both (i) and (ii)).

**Table 2:** Intervention exposure and contamination

	Number (%) of mothers in intervention villages at endline (‘exposure’) *n* = 1254	Number (%) of mothers in control villages at endline (‘contamination’) *n* = 1237
Mother reports that there are active programs promoting institutional delivery in her area and:		
There are any programs that give money to a mother if she delivers in a public health centre^b^	285/1228^a^ (23.2%)	95/1189^a^ (8.0%)
The programs give money to a mother for transportation to a health centre for delivery	231/1228 (18.8%)	97/1189 (8.2%)
The programs give money to CHWs if mothers deliver in a health centre	130/1228 (10.6%)	51/1189 (4.3%)
The programs provide information about the importance of delivering a baby in a health centre	95/1228 (7.7%)	159/1189 (13.4%)
The programs provide transportation to a health centre	380/1228 (30.9%)	194/1189 (16.3%)
Mother reported that she knew she would receive our CCT incentive if she delivered in a health facility^b^	118/1254 (9.4%)	119/1237 (9.6%)
Composite measure of CCT program exposure^c^	342/1254 (27.3%)	195/1237 (15.8%)

*CCT* conditional cash transfer

^a^ 26 women in intervention villages and 48 women in control villages reported they “did not know” and were excluded

^b^ Included in the composite measure

^c^ Mother reported that she knew (i) there were programs that would give her money if she delivered in a health facility or (ii) she would receive our conditional cash transfer (CCT) incentive if she delivered in a health facility

Exposure was lowest in the poorest quintile (10.2%, 17/166 women with known wealth quintile data) compared to the least poor women in quintile five (64.8%, 175/270). 195/1237 (15.8%) of the control group also appeared to have also been exposed (‘contaminated’) (Table 2). Contamination was similarly lowest in the poorest quintile (8.1%, 21/258 women with known wealth quintile data) compared to the least poor women in quintile five (38.8%, 59/152).

### Facility delivery

Baseline facility delivery rates were low, 62.9% (744) in the intervention group and 58.7% (726) in the control group. Overall, facility delivery increased in intervention villages by 14.3% and control villages by 8.4%, adjusted mean difference (AMD) 3.3%; 95% confidence Interval (95% CI) (− 0.14 to 0.21), *p* value 0.685 (Table 3). In the least poor quintile the proportion of women who delivered in a health facility increased in the intervention villages by 29.9% and increased in the control villages by 15.4% (AMD 6.6% [− 0.20 to 0.33], p value 0.602) (Table 3). In the poorest quintile the proportion of women who delivered in a health facility increased in the intervention villages by 22.5% and increased in the control villages by 22.4% (AMD 0.8% [− 0.30 to 0.32], p value 0.953).

**Table 3:** Institutional delivery compared between intervention and control areas by quintile

	Number (%) of mothers in intervention	Number (%) of mothers in control	Crude mean difference (95% CI)	*P* value	Adjusted mean difference^a^ (95% CI)	*P* value
Unrestricted analysis						
Overall	*n* = 2432	*n* = 2469	*n* = 4901		*n* = 3883	
Baseline (*n* = 2441)	744/1182 (62.9%)	726/1236 (58.7%)				
Endline (*n* = 2491)	965/1250 (77.2%)	827/1233 (67.1%)				
Difference	14.3%	8.4%	5.9% (0.01–0.11)	0.027	3.3% (−0.14–0.21)	0.685
Quintile 1 (poorest)			*n* = 888		*n* = 708	
Baseline (*n* = 465)	55/111 (49.6%)	165/354 (46.6%)				
Endline (*n* = 423)	119/165 (72.1%)	178/258 (69.0%)				
Difference	22.5%	22.4%	0.1% (−0.14–0.14)	0.979	0.8% (− 0.30–0.32)	0.953
Quintile 2			*n* = 869		*n* = 753	
Baseline (*n* = 446)	141/220 (64.1%)	125/226 (55.3%)				
Endline (n = 423)	192/254 (75.6%)	105/169 (62.1%)				
Difference	11.5%	6.8%	4.7% (−0.08–0.18)	0.475	5.4% (− 0.14–0.24)	0.541
Quintile 3			*n* = 878		*n* = 801	
Baseline (*n* = 455)	190/290 (65.5%)	99/165 (60.0%)				
Endline (*n* = 423)	149/200 (74.5%)	153/223 (68.6%)				
Difference	9.0%	8.6%	0.4% (−0.12–0.13)	0.954	−3.0% (− 0.27–0.20)	0.781
Quintile 4			*n* = 876		*n* = 780	
Baseline (*n* = 459)	153/239 (64.0%)	161/220 (73.2%)				
Endline (*n* = 417)	142/142 (71.3%)	135/218 (61.9%)				
Difference	7.3%	−11.3%	18.6% (0.06–0.31)	0.003	10.7% (−0.20–0.42)	0.465
Quintile 5			n = 869		*n* = 841	
Baseline (*n* = 447)	150/235 (63.8%)	143/212 (67.5%)				
Endline (*n* = 422)	253/270 (93.7%)	126/152 (82.9%)				
Difference	29.9%	15.4%	14.4% (0.03–0.26)	0.011	6.6% (−0.20–0.33)	0.602
Restricted analysis^b^						
Overall	*n* = 1524	*n* = 2275	*n* = 3799		*n* = 2938	
Baseline (*n* = 2418)	744/1182 (62.9%)	726/1236 (58.7%)				
Endline (*n* = 1381)	291/342 (85.1%)	690/1039 (66.4%)				
Difference	22.2%	7.7%	14.5% (0.08–0.21)	< 0.001	6.1% (− 0.17–0.29)	0.575
Quintile 1 (poorest)	*n* = 128	*n* = 591	*n* = 719		*n* = 542	
Baseline (n = 465)	55/111 (49.6%)	165/354 (46.6%)				
Endline (*n* = 254)	13/17 (76.5%)	165/237 (69.6%)				
Difference	26.9%	23.0%	3.9% (−0.20–0.28)	0.746	−0.7% (− 0.42–0.41)	0.971
Quintile 2	*n* = 238	*n* = 366	*n* = 604		*n* = 491	
Baseline (n = 446)	141/220 (64.1%)	125/226 (55.3%)				
Endline (*n* = 158)	17/18 (94.4%)	89/140 (63.6%)				
Difference	30.3%	8.3%	22.0% (0.06–0.38)	0.007	21.4% (0.07–0.36)	0.008
Quintile 3	*n* = 329	*n* = 352	*n* = 681		*n* = 607	
Baseline (n = 455)	190/290 (65.5%)	99/165 (60.0%)				
Endline (*n* = 226)	28/39 (71.8%)	131/187 (70.1%)				
Difference	6.3%	10.1%	−3.8% (−0.22–0.14)	0.684	−5.8% (−0.35–0.24)	0.673
Quintile 4	*n* = 295	*n* = 408	*n* = 703		*n* = 609	
Baseline (*n* = 459)	153/239 (64.0%)	161/220 (73.2%)				
Endline (*n* = 244)	40/56 (71.4%)	114/188 (60.6%)				
Difference	7.4%	−12.6%	20.0% (0.04–0.36)	0.016	6.3% (−0.30–0.42)	0.709
Quintile 5	*n* = 410	*n* = 305	*n* = 715		*n* = 689	
Baseline (*n* = 447)	150/235 (63.8%)	143/212 (67.5%)				
Endline (*n* = 268)	168/175 (96.0%)	71/93 (76.3%)				
Difference	32.2%	8.8%	23.4% (0.11–0.36)	< 0.001	13.5% (−0.21–0.48)	0.404

^a^Adjusted for clustering by district, maternal age, parity, maternal education, quintile and access to health facilities

^b^Intervention endline population restricted to mothers who reported that she knew (i) there were any programs that would give her money if she delivered in a health facility or (ii) she would receive our CCT incentive if she delivered in a health facility; intervention baseline population not restricted. Control endline population restricted to mothers who reported that she did not know (i) or (ii); control baseline population not restricted

Restricting the analysis to women who were known to have received the intervention (treatment on the treated analysis) had little effect overall (AMD 6.1% [− 0.17 to 0.29], *p* value 0.575) or within each quintile (Table 3).

### Antenatal and postnatal care

The proportion of women who had received at least one ANC visit at baseline was 64.8% (760) in the intervention group and 87.3% (1039) in the control group (Table 4). The proportion of women who had received at least one PNC visit at baseline was much lower; 49.1% (571) in the intervention group and 65.0% (764) in the control group.

**Table 4:** ANC, PNC and CHW home visiting compared between intervention and control areas by quintile

	Number (%) of mothers in intervention villages	Number (%) of mothers in control villages	Crude mean difference (95% CI)	*P* value	Adjusted mean difference^a^ (95% CI)	*P* value
ANC						
Overall	*n* = 2427	*n* = 2425	*n* = 4852		*n* = 3871	
Baseline (*n* = 2363)	760/1173 (64.8%)	1039/1190 (87.3%)				
Endline (*n* = 2489)	957/1254 (76.3%)	729/1235 (59.0%)				
Difference	11.5%	−28.3%	39.8% (0.35–0.45)	< 0.001	45.0% (0.18–0.72)	0.004
Quintile 1 (poorest)	*n* = 273	*n* = 599	*n* = 872		*n* = 706	
Baseline (*n* = 449)	70/107 (65.4%)	288/342 (84.2%)				
Endline (n = 423)	88//166 (53.0%)	101/257 (39.3%)				
Difference	−12.4%	−44.9%	32.5% (0.19–0.46)	< 0.001	43.2% (−0.17–1.03)	0.145
Quintile 2	*n* = 467	*n* = 378	*n* = 845		*n* = 746	
Baseline (*n* = 421)	138/212 (65.1%)	192/209 (91.9%)				
Endline (*n* = 424)	153/255 (60.0%)	74/169 (43.8%)				
Difference	5.1%	−48.1%	43.0% (0.31–0.55)	< 0.001	55.4% (0.10–1.00)	0.021
Quintile 3	*n* = 353	*n* = 384	n = 872		*n* = 800	
Baseline (*n* = 448)	179/287 (62.4%)	151/161 (93.8%)				
Endline (n = 424)	174/201 (86.6%)	143/223 (64.1%)				
Difference	24.2%	−29.7%	53.9% (0.43–0.64)	< 0.001	58.0% (0.23–0.94)	0.004
Quintile 4	*n* = 440	*n* = 435	*n* = 875		n = 780	
Baseline (*n* = 453)	162/240 (67.5%)	171/213 (80.3%)				
Endline (n = 422)	174/200 (87.0%)	144/222 (64.9%)				
Difference	19.5%	−15.4%	34.9% (0.24–0.46)	< 0.001	29.0% (−0.08–0.66)	0.112
Quintile 5	*n* = 508	*n* = 358	*n* = 866		*n* = 839	
Baseline (*n* = 445)	157/238 (66.0%)	183/207 (88.4%)				
Endline (n = 421)	260/270 (96.3%)	125/151 (82.8%)				
Difference	30.3%	−5.6%	35.9% (0.26–0.46)	< 0.001	28.8% (−0.04–0.61)	0.077
PNC						
Overall	*n* = 2415	*n* = 2407	*n* = 4822		*n* = 3850	
Baseline (*n* = 2339)	571/1164 (49.1%)	764/1175 (65.0%)				
Endline (*n* = 2483)	905/1251 (72.3%)	690/1232 (56.0%)				
Difference	23.2%	−9.0%	32.3% (0.27–0.38)	< 0.001	31.8% (− 0.05–0.68)	0.080
Quintile 1 (poorest)	n = 273	*n* = 596	n = 869		*n* = 707	
Baseline (n = 445)	56/107 (52.3%)	241/338 (71.3%)				
Endline (n = 424)	78/166 (47.0%)	99/258 (38.4%)				
Difference	−5.3%	−32.9%	27.6% (0.13–0.42)	< 0.001	37.5% (− 0.38–1.13)	0.296
Quintile 2	n = 467	*n* = 373	*n* = 840		*n* = 743	
Baseline (*n* = 418)	112/212 (52.8%)	145/206 (70.4%)				
Endline (n = 422)	140/255 (54.9%)	73/167 (43.7%)				
Difference	2.1%	−26.7%	28.7% (0.15–0.42)	< 0.001	43.7% (− 0.16–1.04)	0.137
Quintile 3	n = 482	*n* = 383	*n* = 865		*n* = 791	
Baseline (*n* = 444)	142/282 (50.4%)	115/162 (71.0%)				
Endline (n = 421)	163/200 (81.5%)	133/221 (60.2%)				
Difference	31.1%	−10.8%	41.9% (0.30–0.54)	< 0.001	45.8% (−0.47–096)	0.071
Quintile 4	437	*n* = 433	*n* = 870		*n* = 775	
Baseline (n = 448)	120/237 (50.6%)	113/211 (53.6%)				
Endline (n = 422)	177/200 (88.5%)	141/222 (63.5%)				
Difference	37.9%	9.9%	27.9% (0.16–0.40)	< 0.001	21.6% (−0.25–0.68)	0.332
Quintile 5	*n* = 506	*n* = 535	*n* = 859		*n* = 834	
Baseline (*n* = 437)	106/236 (44.9%)	99/201 (49.3%)				
Endline (n = 422)	260/270 (96.3%)	125/152 (82.2%)				
Difference	51.4%	32.9%	18.4% (0.07–0.30)	0.002	8.4% (−0.41–0.58)	0.716
CHW home visiting						
Overall	*n* = 2433	*n* = 2436	*n* = 4869		*n* = 3860	
Baseline (*n* = 2414)	529/1186 (44.6%)	637/1228 (51.9%)				
Endline (*n* = 2455)	519/1247 (41.6%)	338/1208 (28.0%)				
Difference	−3.0%	−23.9%	20.9% (0.15–0.26)	< 0.001	12.2% (−0.27–0.51)	0.508
Quintile 1 (poorest)	*n* = 275	*n* = 611	*n* = 886		n = 707	
Baseline (*n* = 466)	71/110 (64.6%)	219/356 (61.5%)				
Endline (*n* = 420)	19/165 (11.5%)	32/255 (12.6%)				
Difference	−53.1%	−48.9%	−4.1% (−0.16–0.08)	0.511	−1.9% (− 0.76–0.72)	0.954
Quintile 2	*n* = 473	*n* = 392	n = 865		*n* = 750	
Baseline (n = 444)	121/218 (55.5%)	105/226 (46.5%)				
Endline (n = 421)	36/255 (14.1%)	28/166 (16.9%)				
Difference	−41.4%	−29.6%	−11.8% (−0.24- -0.00)	0.049	6.4% (−0.53–0.66)	0.819
Quintile 3	*n* = 489	*n* = 377	n = 866		*n* = 790	
Baseline (*n* = 456)	142/291 (48.8%)	66/165 (40.0%)				
Endline (n = 410)	73/198 (36.9%)	61/212 (28.8%)				
Difference	−11.9%	− 11.2%	−0.7% (−0.14–0.12)	0.916	−2.8% (−0.60–0.55)	0.917
Quintile 4	*n* = 436	*n* = 432	*n* = 868		*n* = 773	
Baseline (n = 448)	92/239 (38.5%)	91/219 (41.6%)				
Endline (n = 410)	92/197 (46.7%)	78/213 (36.6%)				
Difference	8.2%	−5.0%	13.1% (0.00–0.26)	0.050	0.0% (−0.35–0.35)	0.999
Quintile 5	*n* = 509	*n* = 359	n = 868		n = 840	
Baseline (n = 447)	61/239 (25.5%)	111/208 (53.4%)				
Endline (n = 421)	242/270 (89.6%)	101/151 (66.9%)				
Difference	64.1%	13.5%	50.5% (0.38–0.63)	< 0.001	34.5% (−0.18–0.87)	0.175

*ANC* antenatal care, *PNC* postnatal care, *CHW* community health worker

^a^Adjusted for clustering by district, maternal age, parity, maternal education, quintile and access to health facilities

Overall, the proportion of women with at least one ANC visit increased in the intervention villages by 11.5% but decreased in the control villages by 28.3% (AMD 45.0% [0.18 to 0.72], *p* value 0.004) (Table 4). In the least poor quintile, the proportion of women with at least one ANC visit increased by 30.3% in the intervention villages and decreased in the control villages by 5.6% (AMD 28.8% [− 0.04 to 0.61], *p* value 0.077). In contrast, in the poorest quintile, ANC decreased in both the intervention villages (− 12.4%) and the control villages (− 44.9%) (AMD 43.2% [− 0.17 to 1.03] p value 0.145).

Overall, the proportion of women with at least one PNC visit increased in the intervention villages by 23.2% and decreased in the control villages by 9.0% (AMD 31.8% [− 0.05 to 0.68], *p* value 0.080) (Table 4). In the least poor quintile the proportion of women with at least one PNC visit increased in the intervention villages by 51.4% and increased in the control villages by 32.9% (AMD 8.4% [− 0.41 to 0.58] *p* value 0.716). In contrast, in the poorest quintile, PNC decreased in both the intervention (− 5.3%) and the control villages (− 32.9%) (AMD -37.5% [− 0.38 to 1.13] p value 0.296).

### CHW home visiting

Less than half of families received any visits from a CHW at baseline, 44.6% (529) in the intervention group and 51.9% (637) in the control group (Table 4). Overall, CHW home visiting decreased more in the control villages (− 23.9%) compared to the intervention villages (− 3.0%) though the difference did not reach statistical significance (AMD -12.2% [− 0.27 to 0.51], *p* value 0.508) (Table 4).

In the least poor quintile CHW home visiting in the intervention districts increased from 25.5% at baseline to 89.6% at endline (AMD 34.5% [− 0.18 to 0.87]) p value 0.175). However, in the poorest quintile CHW home visiting decreased in both intervention (− 53.1%) and control districts (− 48.9%) (AMD -1.9% [− 0.76 to 0.72] p value 0.954) (Table 4).

### Other analyses

**Fig. 2:**
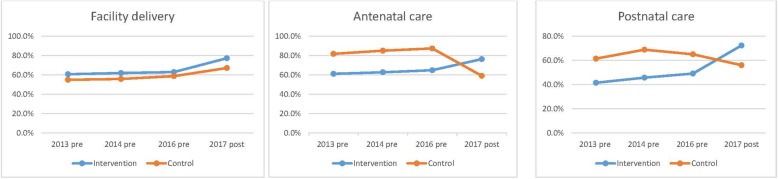
Trends in facility delivery, antenatal care and postnatal care in the study area pre and post intervention

The two key DiD assumptions appeared to hold (Figs. 1 and 2). There were parallel trends in the primary and secondary outcomes pre and post intervention (i.e. we tested for and did not reject the null of parallel trends prior to intervention onset for facility delivery [p value = 0.0748], ANC [p value = 0.0685] and PNC [p value = 0.1029] [Fig. 1]). There was also stable distribution of covariates between intervention and control groups from baseline to endline (Appendix 3). Because 10.9% (525/4929) of mothers had missing data for socio economic status and 360/4929 (7.1%) had missing data for maternal age, we performed sensitivity analyses including the missing data with no change to any outcomes (Appendix 4).

## Discussion

Baseline rates of MCH service use were concerningly low in our study area in rural Afghanistan, only 61% of women delivered in a health facility and 76% received at least one ANC visit. Despite increasing levels of conflict, CCT provided to women aged 16–49 years in our study was associated with increases in maternal and newborn service use and CHW home visiting, though only the change in ANC was statistically significant. There was some evidence of greater effect in the least poor quintile compared to the poorest quintile. Program exposure was low, especially in the poorest quintile.

Overall, there was only a 3% increase in facility delivery associated with the CCT intervention, and this increase was not statistically significant. This contrasts with effects of 10–20% reported in other Asian studies [5, 6]. We feel that low CCT program exposure was an important reason for the modest effect of our intervention. Only 27% of women in intervention areas reported that they had heard about financial incentives or our CCT program. When we performed a ‘per protocol’ analysis (i.e. an analysis restricted to women who knew they would receive money if they delivered in a health facility) [26], the effect size increased though results were still not statistically significant. It is important to understand the reasons for the limited impact of our CCT intervention on institutional delivery (i.e. the incentivized behavior). An indepth analysis of ‘implementation’ ‘process’ data is needed, i.e. to understand the ‘reach’ of our IEC delivery channels including billboards, brochures, CHWs and village leaders. This will be reported in a separate manuscript.

Prior to the onset of the study we conducted detailed formative research and our interviewees (women of reproductive age, male heads of households, village leaders) provided consistent feedback that home visiting from CHWs would be of most use to families. An important part of our planned exposure was home visits from CHWs to raise awareness and to plan transport and assistance for facility delivery and cash transfer. Our CHW data demonstrate the complex nature of working in Afghanistan. Conflict has increased over the 12 months since program implementation [14], and has restricted the movements of the Afghanistan volunteer CHW workforce [28]. In our study, CHW home visits reduced in both intervention and control areas over the intervention period but the decrease was greater in the control areas. This suggests that our CCT intervention may have improved CHW motivation in the intervention areas despite the increasing conflict. Ongoing Afghanistan HMIS surveillance [17] will help to understand further trends. We also feel that our CHWs could be better trained in home visiting as a recent study indicates this is an important way of accessing pregnant and postpartum women in Afghanistan and can significantly improve ANC, facility delivery and PNC [29]. Due to funding constraints we only allocated one day to the CHW training and had to cover many topics from record keeping to IEC messages. Future work must include longer training periods and pre and post training assessments.

We actively monitored impact on ANC and PNC services because we were concerned that their coverage might decrease. However, surprisingly, our intervention was associated with improvements in both ANC and PNC coverage, though only the change in ANC was statistically significant. This is most likely due to the effectiveness of the ANC and PNC messages that were included in our IEC campaigns. Our findings are also consistent with previous studies in Afghanistan reporting the effectiveness of basic ANC and PNC messages communicated by CHWs [29, 30]. Also, in Asian studies, mothers report that they prefer travelling for ANC visits compared to PNC or facility delivery because there is more choice of clinic site and timing [31]. Our formative research indicates that the situation is similar in Afghanistan [9].

Disappointingly the effect of our CCT intervention was consistently lowest in the poorest quintile. The low rate of program exposure in the poorest women was also of concern. Poor uptake of CCT interventions in the most disadvantaged communities is reported in many other studies [3, 31]. The importance of designing CCT programs so they are appropriate to the local context and meet the needs of the poorest families is clearly described [31]. We included remote areas and trained CHW to prioritise the poorest families in their home visiting. However, the CHWs reported difficulties in accessing the poorest families due to lack of trust and the distances they needed to travel. More work is needed in our study area to ensure that our interventions are truly progressive and understand the perspectives of the most disadvantaged families.

We are very aware that quality of health service provision is a driver of demand [32, 33]. At the health facility level our CCT intervention focused on efficiency of payments to the family and CHW. We did implement a HSS package in both the intervention and control health facilities but this focused on training and quality of care and we were not able to increase staffing levels. The mothers who participated in our formative research reported that sometimes no midwives were present to assist with deliveries after hours [9]. In Afghanistan, due to low wages, some doctors and nurses work in private clinics in the afternoons and evenings. However, in our study, these problems were likely to be non-differential across intervention and control groups. Most importantly, the MoPH is working to improve workforce conditions and staffing levels for MCH services.

We encountered other challenges especially conflict reducing access to onsite training of CHWs and community leaders. This delayed the start of the project by six months. Monitoring is a challenge for all programs across Afghanistan due to conflict and mountainous regions [34]. However, in our study we included additional funds for overnight accommodation and for male staff to accompany our largely female data collectors and supervisors. We also included a dedicated independent field worker to monitor the cash transfer.

Our study had some limitations. It was non-randomised. Our study findings can only be generalised to public health facilities because there were no private facilities in our study area. Intervention and control districts were purposively chosen to ensure similar socio economic status of families but there were still baseline differences in socio demographic characteristics in the intervention and control districts. However, ‘difference-in-differences’ analyses were used to account for potentially differing trends in control and intervention areas. We also adjusted for all potential confounding factors though residual confounding was still possible. We were not able to collect cost data from families or perform a cost effectiveness analysis. Outcome data were self-reported and verification was not possible due to project restrictions, however we consider that any recall bias was likely to be non-differential. We also only allowed 12 months for implementation and we are aware that CCT projects can take time to embed. Some women might have only been exposed to the intervention for a limited period before they delivered. However, the CCT message was quite simple so we feel that the duration of intervention was of limited importance. We also consider that exposure rates were underestimated in our study. The intervention was delivered to all family members but the ascertainment of ‘exposure’ data relied solely on maternal self report. Literacy (26.2%) and education rates (15.6%) were low in our study and mothers may not have understood some of the more complex exposure questions. If we had been able to ascertain exposure more accurately we may have been able to show a greater impact ‘per protocol’. 10% of our quintile and 7% of our maternal age data were missing however no change to results were observed when these data were included in the primary and secondary analyses.

Difference-in-differences’ analyses require two main assumptions to be fulfilled: ‘common shocks’ and ‘parallel trends’ [23, 35]. We showed stable distribution of covariates between intervention and control groups from baseline to endline indicating no disparate changes in the intervention compared to the control group. There were also no differential policy shocks or changes between the intervention and control groups during the intervention period. However there has been a major escalation in conflict in both the intervention and control districts over the study period. We feel that that the CCT had an important effect in maintaining ANC, PNC and CHW homevisiting levels in the intervention group and without the CCT program the ANC, PNC and CHW levels would have been the same level as the control group. We consider that facility delivery was preserved in the intervention and control districts as there has been a major focus on facility delivery across Afghanistan from the MoPH. We also tested for and did not reject the null of parallel trends. Though it is important to note that there were some systematic differences between intervention and control groups, and our sample size did not provide enough statistical precision to conclusively reject the null.

Strengths included our prospective community and population based design, large sample size and high response rate (94%). We had an intensive data quality and supervision plan which included scheduled and unscheduled revisits. Overall, our study demonstrated that a CCT program provided to women aged 16–49 years can be implemented in a highly conservative conflict affected population and can include fathers and male heads of households as well as female family members. It is likely that if we can improve our focus on the poorest families, CCT will have important impacts on facility delivery, ANC and PNC.

## Conclusions

Almost 400 million women and children are estimated to live in conflict-affected areas globally and these numbers are increasing [36]. Our innovative study demonstrates the important ‘proof of principle’ that CCT for mothers and newborns can be implemented in emergency situations and fragile states. There is an imperative to implement and evaluate novel and ‘outside the box’ interventions for the mothers and children that live in deprived and conflict affected communities across the world. We encourage other governments and researchers to also implement and evaluate CCT in conflict affected areas.

## Appendix 1

**Table 5:** Characteristics of intervention and control districts

	Intervention districts (*n* = 6)	Control districts (*n* = 6)
Population		
Total population	544,191	657,906
Mean (sd) population	10,884 (11,824)	15,300 (17,418)
Total number of pregnant women	21,767	26,316
Mean (sd) number of pregnant women per district	3628 (1857)	4386 (2231)
Total number of children under five years	108,838	131,581
Mean (sd) number of children under five years per district	181,40 (9282)	21,930 (11,154)
Sociodemographics^a^		
Mean (sd) % poorest wealth quintile	843 (917)	1012 (965)
Mean (sd) % no female education	1638 (2004)	2380 (3052)
Access^a^		
% Remote	100%	100%
% Mountainous	86.0%	81.4%
% High security risk	24.0%	34.9%
Health services^a^		
Total number of fixed health facilities (SHC, BHC, CHC, DH)	50	43
Number of sub health centres	16	12
Mean (sd) sub health centres per district	4.0 (4.2)	3.0 (1.8)
Mean (sd) population per sub health centre	24,481 (21,330)	25,830 (25,427)
Number of basic health centres	21	18
Mean (sd) basic health centres per district	3.5 (1.4)	3.0 (1.8)
Mean (sd) population per basic health centre	58,632 (40055)	50,430 (42355)
Number of comprehensive health centres	4	6
Mean (sd) comprehensive health centres per district	1.0 (0.0)	1.2 (0.4)
Mean (sd) population per comprehensive health centre	17,851 (16,209)	33,140 (32,561)
Number of district hospitals	1	2
Mean (sd) district hospitals per district	–	1.0 (0.0)
Mean (sd) population per district hospital	12,472 (0)	34,048 (27,969)
Health facility density (population per health facility)	10,268	15,300
Health facility density (pregnant women and children under five population per health facility)	2612	3672
Number of vaccination outreach services	4	6
Number of mobile health teams	4	5
Number of community health workers	7	8
Number of mini ambulances	1479	1353

*sd* standard deviation, *SHC* sub health centre, *BHC* basic health centre, *CHC* comprehensive health centre, *DH* district hospital

^a^ See Appendix 2 for definitions

## Appendix 2

**Table 6:** Definitions of terms and covariates used in the study

Health services	
Sub health centre (SHC)	Targeted to whole population Staffed by generalist nurse, vaccinator, midwife Provide antenatal care, postnatal care, growth monitoring, vaccination and integrated management of childhood illness (IMCI) clinics but no delivery care or emergency care (emergency cases are stabilised and referred to BHC, CHC or DH) [15].
Basic health centre (BHC)	Targeted to whole population Staffed by generalist nurse, vaccinator, midwife Provide services as above, but also assistance to normal deliveries (emergency cases are stabilised and referred to BHC, CHC or DH) [15].
Comprehensive health centre (CHC)	Targeted to whole population Staffed by generalist nurse, vaccinator, midwife, doctor Provide services as above, but also basic emergency obstetric care from a midwife which includes manual removal of placenta and retained products, blood transfusion, basic laboratory services. Also provides vaccination outreach (one outreach service per CHC) [15]
District hospital (DH)	Targeted to whole population Staffed by generalist nurse, vaccinator, midwife, doctor Provide services as above, but also comprehensive emergency obstetric care from a doctor or nurse which includes surgery, anaesthesia and Caesarean section. No outreach vaccination service [15].
Health facility density	Population per health facility
Socio-demographics	
Poorest wealth quintile	Women scored in asset index as being in the lowest 20% of the population [20].
No female education	Women with no formal education [20].
Any female	
Access	
Remote	District centre more than 2 h by any form of transport from provincial capital [14].
Mountainous	More than 1800 km elevation at highest point of district [14].
High security risk	Use of armed force between warring parties in a conflict dyad, state-based or non-state, resulting in deaths). 25 deaths or less in the previous 12 months is categorised as low intensity security risk, 25–100 is categorised as moderate intensity security risk and 100+ is categorised as high intensity security risk [13].

*SHC* sub health centre, *BHC* basic health centre, *CHC* comprehensive health centre, *DH* district hospital

## Appendix 3

**Table 7:** Difference in difference analysis of sociodemographic characteristics of women included in the study compared between intervention and control villages

	Number (%) of mothers in intervention villages	Number (%) of mothers in control villages	Difference in intervention compared to control at baseline	Number (%) of mothers in intervention villages	Number (%) of mothers in control villages	Difference in intervention compared to control at endline	Difference in difference analysis between baseline and endline Crude mean difference (95% CI)
	Baseline	Baseline		Endline	Endline		
	(*n* = 1199)	(*n* = 1242)		(*n* = 1254)	(*n* = 1237)		
Province							
Bamyan	421 (35.1%)	413 (33.3%)	− 1.8%	403 (32.1%)	403 (32.6%)	0.5%	−2.3% (− 0.08–0.03)
Badghis	387 (32.3%)	412 (33.2%)	0.9%	400 (31.9%)	404 (32.7%)	0.8%	0.1% (−0.05–0.05)
Kandahar	391 (32.6%)	417 (33.6%)	1.0%	451 (36.0%)	430 (34.8%)	−1.2%	2.2% (− 0.03–0.07)
Maternal education							
Primary school (year 1–6)	38 (3.2%)	74 (6.0%)	2.8%	46 (3.7%)	86 (7.0%)	3.3%	−0.4% (− 0.03–0.02)
Secondary school (year 7–9)	33 (2.8%)	40 (3.2%)	0.4%	34 (2.7%)	38 (3.1%)	0.4%	0.1% (−0.02–0.02)
High school (year 10–12)	32 (2.7%)	57 (4.6%)	1.9%	72 (5.7%)	73 (5.9%)	0.2%	1.7% (−0.01–0.04)
Advanced education (year 12+)	14 (1.2%)	22 (1.8%)	0.6%	20 (1.6%)	49 (4.0%)	2.4%	−1.8% (−0.03- -0.001)
No education	1082 (90.2%)	1038 (83.6%)	−6.6%	1057 (84.3%)	983 (79.5%)	−4.8%	−1.8% (−0.06–0.02)
Literacy							
Literate	223 (18.6%)	279 (22.5%)	3.9%	412 (32.8%)	382 (30.9%)	−1.9%	5.8% (0.01–0.11)
Not literate	976 (81.4%)	963 (77.5%)	−3.9%	842 (67.2%)	855 (69.1%)	1.9%	−5.8% (−0.11- -0.01)
Mother’s age							
16–24 years	469 (39.1%)	455 (36.6%)	−2.5%	496 (39.6%)	391 (31.6%)	−8.0%	5.4% (−0.01–0.11)
25–29 years	407 (33.9%)	346 (27.9%)	−6.0%	307 (24.5%)	386 (31.2%)	6.7%	−12.8% (−0.17–0.07)
30–39 years	213 (17.8%)	145 (11.7%)	−6.1%	364 (29.0%)	282 (22.8%)	−6.2%	0.1% (−0.04–0.05)
40 years or older	108 (9.0%)	70 (5.6%)	−3.4%	86 (6.9%)	47 (3.8%)	3.1%	−0.3% (− 0.03–0.02)
Mother’s parity							
1	112 (9.3%)	96 (7.7%)	−1.6%	138 (11.0%)	188 (15.2%)	4.2%	−5.8% (−0.09–0.02)
2	161 (13.4%)	183 (14.7%)	1.3%	165 (13.2%)	238 (19.2%)	6.0%	−4.7% (− 0.09–0.01)
3	209 (17.4%)	242 (19.5%)	2.1%	160 (12.8%)	208 (16.8%)	4.0%	−2.0% (− 0.06–0.02)
4	174 (14.5%)	209 (16.8%)	2.3%	203 (16.2%)	150 (12.1%)	−4.1%	6.4% (−0.02–0.10)
5 or more	543 (45.3%)	512 (41.2%)	−4.1%	588 (46.9%)	453 (36.6%)	−10.3%	6.2% (−0.01–0.12)
Wealth quintile							
Quintile 1 (poorest)	111 (9.3%)	358 (28.8%)	19.5%	166 (13.2%)	258 (20.9%)	7.7%	11.9% (−0.08–0.16)
Quintile 2	223 (18.6%)	228 (18.4%)	−0.2%	255 (20.3%)	169 (13.7%)	−6.6%	6.4% (0.02–0.11)
Quintile 3	294 (24.5%)	165 (13.3%)	−11.2%	201 (16.0%)	223 (18.0%)	2.0%	−13.2% (0.18- -0.09)
Quintile 4	241 (20.1%)	220 (17.7%)	−2.4%	200 (16.0%)	222 (18.0%)	2.0%	−4.3% (0.09- -0.01)
Quintile 5 (least poor)	239 (19.9%)	212 (17.1%)	−2.8%	270 (21.5%)	152 (12.3%)	−9.2%	6.4% (−0.02–0.11)
Access to any health facility							
Less than 30 mins	422 (35.2%)	299 (24.1%)	−11.1%	419 (33.4%)	376 (30.4%)	−3.0%	−8.1% (0.13- -0.03)
30 mins to 1 h	405 (33.8%)	595 (47.9%)	14.1%	509 (40.6%)	531 (42.9%)	2.3%	11.8% (−0.06–0.17)
1–2 h	172 (14.4%)	183 (14.7%)	0.3%	236 (18.8%)	211 (17.1%)	−1.7%	2.1% (− 0.02–0.06)
2 h to half a day	124 (10.3%)	25 (2.0%)	−8.3%	65 (5.2%)	35 (2.8%)	−2.4%	−6.1% (0.09- -0.04)
More than half a day	29 (2.4%)	10 (0.8%)	−1.6%	1 (0.1%)	54 (4.4%)	4.3%	−6.0 (0.08- -0.04)

## Appendix 4

**Table 8:** Sensitivity analysis including missing data

	Number (%) of mothers in intervention villages	Number (%) of mothers in control villages	Crude mean difference (95% CI)	*P* value	Adjusted mean difference^a^ (95% CI)	*P* value
Facility delivery quintile 3						
Excluding missing data			*n* = 878		*n* = 801	
Baseline (*n* = 455)	190/290 (65.5%)	99/165 (60.0%)				
Endline (*n* = 423)	149/200 (74.5%)	153/223 (68.6%)				
Difference	9.0%	8.6%	0.4% (−0.12–0.13)	0.954	−3.0% (− 0.27–0.20)	0.781
Including missing data	*n* = 739	*n* = 660	*n* = 1399		*n* = 1256	
Baseline (*n* = 621)	245/377 (65.0%)	132/224 (58.9%)				
Endline (*n* = 798)	259/362 (71.6%)	283/436 (64.9%)				
Difference	6.6%	6.0%	0.6% (−0.98–0.11)	0.912	−0.5% (− 0.18–0.17)	0.951
Excluding missing data	n = 353	n = 384	n = 872		*n* = 800	
Baseline (*n* = 448)	179/287 (62.4%)	151/161 (93.8%)				
Endline (*n* = 424)	174/201 (86.6%)	143/223 (64.1%)				
Difference	24.2%	−29.7%	53.9% (0.43–0.64)	< 0.001	58.0% (0.23–0.94)	0.004
Including missing data	n = 739	*n* = 655	*n* = 1394		*n* = 1256	
Baseline (*n* = 595)	233/376 (62.0%)	205/219 (93.6%)				
Endline (*n* = 799)	282/363 (77.7%)	285/436 (65.4%)				
Difference	15.7%	−28.2%	43.9% (0.35–0.53)	< 0.001	49.4% (0.21–0.77)	0.003
PNC quintile 3						
Excluding missing data	n = 482	n = 383	n = 865		n = 791	
Baseline (*n* = 444)	142/282 (50.4%)	115/162 (71.0%)				
Endline (n = 421)	163/200 (81.5%)	133/221 (60.2%)				
Difference	31.1%	−10.8%	41.9% (0.30–0.54)	< 0.001	45.8% (−0.47–096)	0.071
Including missing data	*n* = 732	*n* = 652	*n* = 1384		*n* = 1245	
Baseline (*n* = 591)	177/372 (47.6%)	166/219 (75.8%)				
Endline (*n* = 793)	250/360 (69.4%)	252/433 (58.2%)				
Difference	21.8%	−17.6%	39.4% (0.29–0.50)	< 0.001	49.8% (−0.01–1.00)	0.054
CHW home visiting quintile 3						
Excluding missing data	*n* = 489	*n* = 377	*n* = 866		*n* = 790	
Baseline (n = 456)	142/291 (48.8%)	66/165 (40.0%)				
Endline (*n* = 410)	73/198 (36.9%)	61/212 (28.8%)				
Difference	−11.9%	−11.2%	−0.7% (−0.14–0.12)	0.916	−2.8% (− 0.60–0.55)	0.917
Including missing data	*n* = 740	*n* = 642	*n* = 1382		*n* = 1239	
Baseline (*n* = 599)	184/380 (48.4%)	111/219 (50.7%)				
Endline (*n* = 783)	130/360 (36.1%)	99/423 (23.4%)				
Difference	−12.3%	−27.3%	15.0% (0.04–0.25)	0.005	16.0% (−0.49–0.81)	0.596

^a^Adjusted for clustering by district, maternal age, parity, maternal education, quintile and access to health facilities

## Abbreviations

95% CI: 95% confidence intervals
AMD: Adjusted mean difference
ANC: Antenatal care
BEmONC: Basic and emergency obstetric and newborn care
CCT: Conditional cash transfer
CHS: Community health supervisor
CHW: Community health worker
DiD: Difference-in-differences
ENC: Essential newborn care
HMIS: Health management information system
HSS: Health systems strengthening
IEC: Information, education, communication
MCH: Maternal and child health
MD: Mean difference
MoPH: Ministry of Public Health
ORS: Oral rehydration therapy
PNC: Postnatal care
